# Examination of the SUPPS-P Impulsive Behavior Scale among Male and Female Youth: Psychometrics and Invariance

**DOI:** 10.3390/children8040283

**Published:** 2021-04-07

**Authors:** Pedro Pechorro, Rebecca Revilla, Victor H. Palma, Cristina Nunes, Cátia Martins, Melissa A. Cyders

**Affiliations:** 1 School of Psychology, University of Minho, Campus de Gualtar, 4710-057 Braga, Portugal; ppechorro@gmail.com; 2 Department of Psychology, The University of Alabama, 505 Hackberry Lane, 348 Gordan Palmer Hall, Tuscaloosa, AL 35401, USA; rrevilla@crimson.ua.edu; 3 CinTurs, Universidade do Algarve, Campus de Gambelas, 8005-139 Faro, Portugal; victorhugopalma@sapo.pt; 4 Psychology Research Centre (CIP), Universidade do Algarve, Campus de Gambelas, 8005-139 Faro, Portugal; csmartins@ualg.pt; 5 Department of Psychology, School of Science, Indiana University Purdue University Indianapolis, 402 N. Blackford St., LD 124, Indianapolis, IN 46202, USA; mcyders@iu.edu

**Keywords:** assessment, measurement invariance, SUPPS-P impulsive behavior, validation, youth

## Abstract

The UPPS-P Impulsive Behavior Scale is one of the most used and easily administered self-report measures of impulsive traits. The main objective of this study was to examine the psychometric properties of the shorter SUPPS-P scale among a school sample of 470 youth (*M*_age_ = 15.89 years, *SD* = 1.00) from Portugal, subdivided into males (*n* = 257, *M_age_* = 15.97 years, *SD* = 0.98) and females (*n* = 213, *M_age_* = 15.79 years, *SD* = 1.03). Confirmatory factor analysis results revealed that the latent five-factor structure (i.e., Negative urgency, Lack of perseverance, Lack of premeditation, Sensation seeking, and Positive urgency) obtained adequate fit and strong measurement invariance demonstrated across sex. The SUPPS-P scale also demonstrated satisfactory psychometric properties in terms of internal consistency, discriminant and convergent (e.g., with measures of youth delinquency, aggression) validities, and criterion-related validity (e.g., with crime seriousness). Findings support the use of the SUPPS-P scale in youth. Given the importance of adolescence as a critical period characterized by increases in impulsive behaviors, having a short, valid, reliable, and easily administered assessment of impulsive tendencies is important and clinically impactful.

## 1. Introduction

Adolescence is the developmental time period most characterized by an increase in impulsive behavior [1,2]. According to the dual-systems model, this increase is attributed to developmental changes in two interacting neurobiological systems: a heightened sensitivity to rewards in the socioemotional system (e.g., limbic regions) and a slowly maturing cognitive control system (e.g., prefrontal regions) [3,4]. Consequently, adolescents are more vulnerable to engaging in harmful risk-taking behaviors, such as substance use, criminal activity, and unprotected sexual behavior [5]. For these reasons, having a valid measure that can quickly and accurately assess impulsive tendencies among youth allows for early identification of risk and intervention to avoid negative consequences associated with such maladaptive behaviors.

The UPPS-P Impulsive Behavior Scale is one of the most commonly used and easily administered self-report measures of impulsive traits. The measure was created by Whiteside and Lynam [6], who used factor analysis to identify four facets of personality that influence impulsive behavior: Urgency is the tendency to act rashly when experiencing extreme negative emotion. Lack of premeditation is the tendency to act without thinking or reflecting on negative consequences that may result from the act. Lack of perseverance is the tendency to lose focus and discontinue a task if it is boring or difficult. Sensation seeking refers to the tendency to seek out exciting and new activities that may coincide with danger. Cyders and colleagues [7,8] proposed that urgency consisted of both negative urgency (originally included by Whiteside and Lynam) [6] and positive urgency, which is a tendency to act rashly when experiencing extreme positive emotion. The addition of positive urgency as the fifth facet of impulsive behavior is reflected in the UPPS-P Impulsive Behavior Scale [9]. Each of these facets is theorized to have unique relationships with important risk outcomes, and this has been supported by the literature using the UPPS-P (for example) [10,11,12]. Thus, separating impulsivity into different facets can improve our prediction of risk outcomes for high-risk youth.

Due to the long length (59 items) and time (approximately 15 min) needed to complete the UPPS-P, Lynam [13] created a short form version of this scale. The SUPPS-P has a total of 20 items with four items belonging to each of the five subscales. An examination of the SUPPS-P found the measure to be valid and reliable, while a confirmatory factor analysis (CFA) showed a five-factor model and a three-factor hierarchical model to be consistent with the original factor structure of the UPPS-P [14]. Thus, the SUPPS-P offers significant time savings without sacrificing reliability and validity of the constructs measured. Short versions of the UPPS-P have been validated in other languages including French, Spanish, Italian, Arabic, Swedish, and Korean and studies on these versions suggest a five-factor interrelated model provides the best fit to the data, along with good to acceptable internal consistency and external validity [15,16,17,18,19,20]. Currently, there is no validation of a Portuguese-language version of the SUPPS-P. However, it is worth mentioning that Dias, Chóliz, and Cadime [21] previously translated a short version of the UPPS, which did not include the positive urgency facet, into Portuguese to assess its psychometric properties and study measurement invariance between Portuguese and Spanish college-aged students. Unfortunately, only partial measurement invariance was supported across the Spanish and Portuguese samples, and the four-factor model supported in the Spanish sample could not be replicated in the Portuguese sample data (although removing two items improved the fit for both samples).

There are important sex differences in impulsivity (see a review by Cross, Coping, and Campbell) [22] that can exacerbate risk-taking behaviors in adolescents (for example) [23]. However, before researchers can establish that one sex shows greater impulsivity than the other, it is necessary to first establish that the scale being used to measure impulsivity functions the same across the sexes. Cyders [24] thus examined the full UPPS-P factor structure and invariance measurement through adult men and women and concluded that (1) the five-factor model of the UPPS-P is valid across males and females, (2) males reported higher sensation seeking and positive urgency than females, and (3) UPPS-P traits and risk outcomes relationship did not differ across males and females. Argyriou, Um, Wu, and Cyders [25] later examined measurement invariance of the UPPS-P within the adult life span, mostly replicating and extending findings by Cyders [24] in a more diverse sample: Multigroup analysis showed full invariance of the five-factor model across sex, higher sensation seeking in males, and no difference in how UPPS-P traits relate to risk-taking outcomes across sex. However, most of the work in this area has focused on adult life span. Thus, using the SUPPS-P to make determination of sex differences in adolescents requires examination of measurement invariance in this group. Unfortunately, we are not aware of any previous studies examining the cross-sex measurement invariance of the SUPPS-P among adolescents in North American or European samples.

The main aim of the current study is to translate the SUPPS-P into Portuguese and to examine the psychometric properties and measurement invariance of this measure in school-based male and female Portuguese youth. To achieve this goal, we have six aims. The first aim is to examine the factor structure of the SUPPS-P scale using CFA. Based on prior work, we predict that a five-factor model will best fit the data for the full sample. The second aim is to test measurement invariance by examining how the psychometric properties of the scale might differ by sex (male and female youth). We also use a multigroup CFA to investigate if the factor structure and the factor loadings vary across sex. The third aim is to examine internal consistency of the scale, measured by Cronbach’s alpha and Omega coefficient, across males and females. The fourth aim is to examine convergent validity by identifying the relationship among the SUPPS-P traits and measures of youth psychopathic/dark triad traits, delinquency, conduct disorder and peer aggression as part of the validation process. The last aim is to examine criterion-related and discriminant validity by identifying the relationship among the SUPPS-P traits, a self-control scale, and a crime seriousness index.

We predict that the SUPPS-P will present: (1) a latent five-factor structure and measurement invariance; (2) adequate internal consistency (Cronbach´s alpha and Omega coefficient); (3) convergent validity (e.g., with youth psychopathic traits, delinquency, aggression), and discriminant validity (with a self-control scale); (4) criterion validity (e.g., with crime seriousness). Establishing the validity and reliability of this scale is a critical step for determining whether the translated SUPPS-P scale can be used in Portuguese youth. Additionally, comparisons between male and female youth cannot be made until it has been documented that the scale measures these traits in the same way and to the same degree across these groups.

## 2. Materials and Methods

### 2.1. Participants

Youth participants were collected from the Portuguese public school system (*N* = 470, *M* = 15.89 years, *SD* = 1.00, age range = 14–18). The sample was split into two groups—male group (*n* = 257, *M* = 15.97 years, *SD* = 0.98, age range = 14–18) and female group (*n* = 213, *M* = 15.79 years, *SD* = 1.03, age range = 14–18). Most were Portuguese citizens (88.3%) from urban areas who had completed, on average, 9 years of education (*M* = 8.96, *SD* = 0.95). No significant differences emerged between the two groups with respect to years of age (*F* = 7.786, *p* = 0.052), education (*F* = 0.534, *p* = 0.47), or socioeconomic status of the parents (SES) (*U* = 26336, *p* = 0.45).

### 2.2. Measures

*SUPPS-P Impulsivity Scale* (SUPSS-P) [13]. This instrument is a short self-report questionnaire designed to measure impulsivity derived from the original UPPS-P. It encompasses five factors with four items each (total of 20 items): Negative urgency, Lack of perseverance, Lack of premeditation, Sensation seeking, and Positive urgency. All items are formatted as 4-point Likert scales with anchors 1 (=Strongly agree) and 4 (=Strongly disagree). After reversing the appropriate items, factor scores are attained by summing the respective items. An elevated prevalence of impulsivity is reflected in higher scores. Adequate psychometric properties were reported in previous studies (e.g., internal consistency) [14]. The present study used the Portuguese version of the SUPPS-P. Reliability results are given below in the Results section.

*Youth Psychopathic Traits Inventory Triarchic—Short* (YPI-Tri-S) [26]. This instrument is a brief self-report measure with relevant importance to the Triarchic model of psychopathy construct [27]. It encompasses three factors with seven items each (total of 21 items), namely: Boldness, Disinhibition, and Meanness. All items are formatted as 4-point Likert scales with anchors 0 (=Does not apply at all) and 3 (=Applies very well). One can obtain factor scores (by summing all items scores) and also a total score (through the sum of all items scores). An elevated prevalence of these triarchic psychopathic traits is reflected in higher scores. The reliability for this study, estimated by alpha, was: Boldness = 0.83, Disinhibition = 0.87, Meanness = 0.91, and YPI-Tri-S total = 0.95. Boldness correlated at 0.83 (*p* < 0.001) with Disinhibition and at 0.80 (*p* < 0.001) with Meanness, and Disinhibition correlated at 0.84 (*p* < 0.001) with Meanness.

*Dirty Dozen* (DD) [28]. This instrument is a short self-report measure based on the Dark Triad construct of personality, which taps unpleasant personality traits associated mainly with social behaviors (e.g., self-centering, manipulation). It encompasses three factors with four items each (total of 12 items), namely: Narcissism, Machiavellianism, and Psychopathy. All DD items in the current study were formatted as 5-point Likert scales with anchors 1 (=Strongly agree) and 5 (=Strongly disagree). Factor scores are attained by summing the respective items. An elevated prevalence of these dark traits is reflected in higher scores. The DD Portuguese version for adolescents was employed in the current study [29]. The reliability for this study was: Narcissism = 0.86, Machiavellianism = 0.86, Psychopathy = 0.94, and DD total = 0.93. Narcissism correlated at 0.59 (*p* < 0.001) with Machiavellianism and at 0.54 (*p* < 0.001) with Psychopathy, and Machiavellianism correlated at 0.72 (*p* < 0.001) with Psychopathy.

*Peer Conflict Scale-20* (PCS-20) [30]. This self-report instrument is a PCS brief version developed by Marsee and colleagues [31] to assess aggression. It encompasses four factors with five items each (total of 20 items), namely: Reactive-Relational, Reactive-Overt, Proactive-Relational, and Proactive-Overt. All items are formatted as 4-point Likert scales with anchors 0 (=Not at all true) and 3 (=Definitely true). Four subscale scores are obtained by summing respective items. An elevated prevalence of aggression levels is reflected in higher scores. Previous studies showed good reliability for all subscales and total PCS-20 scores (for example) [32]. The PCS-20 Portuguese version for adolescents was employed in the current study [33]. The reliability for this study was: Reactive-Relational = 0.82, Reactive-Overt = 0.95, Proactive-Relational = 0.87, Proactive-Overt = 0.95, and PCS-20 total = 0.96. Reactive-Relational correlated at 0.53 (*p* < 0.001) with Reactive-Overt, at 0.59 (*p* < 0.001) with Proactive-Relational and at 0.54 (*p* < 0.001) with Proactive-Overt; Reactive-Overt, correlated at 0.64 (*p* < 0.001) with Proactive-Relational and at 0.84 (*p* < 0.001) with Proactive-Overt; Proactive-Relational correlated at 0.56 (*p* < 0.001) with Proactive-Overt.

*Adolescent Health Self-Report Delinquency* (AHSRD) [34,35]. This instrument is composed of items originating from the National Longitudinal Study of Adolescent Health [34]. It encompasses two factors, violent delinquency and nonviolent delinquency, with seven items and ten items, respectively (a total of 17 items). This instrument assesses the occurrence of self-report delinquency acts during/in the last 12 months. All items are formatted as 4-point Likert scales with anchors 0 (=None) and 3 (=Five or more times). It is scored by summing the respective items of each of the two factors, and the use of a total score is also recommended. An elevated prevalence of delinquency acts is reflected in higher scores. The reliability for this study was: Violent = 0.97, Nonviolent = 0.93, and AHSRD total = 0.97. Violent correlated at 0.78 (*p* < 0.001) with Nonviolent.

*Conduct Disorder Screener* (CDS) [36]. This instrument is a brief self-report questionnaire created to screen for youth with conduct disorder. It is composed of only one factor with a total of six items representative of the Conduct Disorder diagnosis of the DSM-IV [37]. All items are formatted as 4-point Likert scales with anchors 1 (=Rarely or none of the time) and 4 (=Most or all of the time). It is scored by summing the items to obtain the total score. An elevated prevalence of conduct disorder symptoms is reflected in higher scores. The CDS Portuguese version for adolescents was employed in the current study [38]. The reliability for this study was 0.92.

*Brief Self-Control Scale* (BSCS) [39]. This instrument is a brief measure of the self-report construct that was derived of the SCS. It is composed of only one factor with a total of thirteen items. All items are formatted as 5-point Likert scales with anchors 1 (=Not at all like me) and 5 (=Very much like me). It is scored by summing the items to obtain the total score. An elevated prevalence of self-control is reflected in higher scores. The BSCS Portuguese version for adolescents was employed in the current study [40]. The reliability for this study was 0.94.

A self-report version of the *General Delinquency Seriousness Classification* (GDSC) [41] adapted to the Portuguese reality was employed to classify criminal behaviors reported by participants, with items formatted as 6-point Likert scales with anchors 0 (=no delinquency acts reported) and 5 (=two or more acts of serious delinquency reported—e.g., car theft, breaking and entering).

A self-report questionnaire designed to measure sociodemographic variables (e.g., nationality, sex, SES) [42] was employed to complement the psychometric measures described above.

### 2.3. Procedures

The usual method employing back-translation was used to translate the SUPPS-P to the Portuguese language (see van de Vijver) [43]. The first author and the third author translated the instrument into Portuguese. An English speaking translator that was also a native English speaker did the back-translation. To ensure that no substantial differences between the two versions of the SUPPS-P existed, some improvements were made. Subsequently, the translation was submitted to a pilot test, with the results being declared good (Portuguese translation available upon contact to the first or third authors).

The Ministry of Education of Portugal (General Directorate of Education—DGE) gave permission to assess participants from schools of southern Portugal (namely, the Algarve, Lisbon, and Alentejo regions) (Code: 0618500001). This study complied with the DGE research ethics guidelines. After being briefed about the aims and features of the present study, participants were informed that collaboration was strictly voluntary. A few of them declined to participate or were later excluded from participating (e.g., some were reluctant to participate, some were not given permission from their parents/tutors, others did not properly understand the Portuguese language), yet the participation rate was around 92%. No compensation was given for participating. The measures were administered to small groups of participants or individually.

IBM SPSS Statistics v26 [44] and EQS v6.4 [45] software was used to conduct data analysis. Confirmatory factor analyses (CFAs) employed covariance matrices and Maximum Likelihood (ML) robust methods [46]. Fit indices: SB*χ*^2^ (Satorra-Bentler Chi-square), *df* (degrees of freedom), CFI (Comparative Fit Index), RMSEA (Root Mean Square Error of Approximation), IFI (Incremental Fit Index), and AIC (Akaike Information Criterion). For an adequate fit, we considered the following criteria: SBχ^2^/*df* < 3, CFI and IFI > 0.90, RMSEA < 0.08, and lowest AIC; and for a good fit: SB*χ*^2^/*df* < 2, CFI-IFI > 0.95, RMSEA (90% CI) < 0.06, lowest AIC. A loading level ≥ 0.30 was used to retain items [47].

We tested several different models: an initial one-factor model that included all the items load on a single latent factor; a model comprising intercorrelated five-factors with items loading on the correct factors; a model where a five-factor model second order higher factor was used; and a model that examined a bifactor model (general factor and five subordinate factors).

When necessary, modification indexes (MI) were considered when examining model fits. However, none were used to improve the models. Across sex, to test the measurement invariance, we performed multiple group CFAs using the following criteria: changes in CFI (ΔCFI < 0.01), in RMSEA (ΔRMSEA < 0.015), and SB*χ*^2^ test non-significant [48].

Pearson correlations and Spearman correlations were considered high if above 0.50, low if below 0.20, moderate if in between [49]. ANOVAs with the respective effect size (partial Eta squared—*η_p_*^2^) were employed to compare groups [50]. Reliability was examined with Cronbach’s alpha, and also with omega (adequate if above 0.70) to better estimate true reliability [51]. Reliability was also examined with corrected item-total correlation ranges (CITCR; adequate if above 0.20) and mean inter-item correlations (MIIC; adequate if within the 0.15–0.50 range) [52].

## 3. Results

We began our examination of the SUPPS-P focusing on its latent factor structure. Table 1 displays the goodness of fit indices obtained for the different CFA models. The five-factor was considered the best fitting model across the male and female samples.

The item loadings for the five-factor model of the SUPPS-P across the two samples are displayed in Table 2.

Next, we tested for measurement invariance of the SUPPS-P across sex (males versus females) (Table 3). Strong invariance held because the ΔCFI between the models was below the 0.01 cutoff and the ΔRMSEA was below the 0.015 cutoff. This indicates that the constraints that were specified indeed hold, and there is equivalence across sex.

The correlations between the SUPPS-P factors were always positive, moderate to high, and statistically significant among the male and female school samples, in accordance with what was expected (Table 4).

Table 5 presents the internal consistency of the SUPPS-P estimated using several parameters, namely Cronbach’s alphas and Omega coefficients, but also corrected item-total correlations and mean inter-item correlations. Mostly, the values ranged from adequate to good for both the male and female school samples.

Table 6 shows SUPPS-P convergent, discriminant, and criterion-related validities. The convergent validity with triarchic psychopathic traits (YPI-Tri-S), dark triad traits of personality (DD), peer aggression (PCS-20), delinquency (AHSRD), and conduct disorder symptoms (CDS) generally demonstrated the expected positive significant correlations. On the other hand, the discriminant validity was examined with the BSCS, mainly revealing the expected negative significant correlations. Finally, the criterion-related validity conducted with the General Delinquency Seriousness Classification (GDSC) revealed positive significant correlations in accordance with what was expected.

## 4. Discussion

The current study was the first to examine the SUPPS-P among a school sample of Portuguese youth, while testing for measurement invariance across males and females. CFAs showed that the five-factor first-order model presented the best fit when compared to the other models we tested. Other studies [24,25], have obtained support for the five-factor model based on culturally distinct samples. It is important to mention that item 16 did not reach the 0.30 minimum recommended cutoff, but we decided not to remove it because it came very close and, because the scale is used so widely, we were hesitant to modify the SUPPS-P unless it was absolutely necessary to do so.

With regard to measurement invariance across sex, the findings corroborated the presence of weak and strong invariance. This suggests the models share a substantial degree of equivalence across males and females, allowing for unbiased group mean comparisons (for example) [48]. Our study using the SUPPS-P among youth was the first study we are aware of investigating cross-sex invariance using this promising measure. Although the literature tends to accept that males tend to behave more impulsively when compared to females (see, e.g., Cross and colleagues [22]), some investigations examining other impulsivity measures (e.g., Barratt Impulsiveness Scale, BIS-11) did not find significant sex differences [53,54]. Such comparisons cannot be validly made without establishing cross-sex invariance. The SUPPS-P can be used to compare impulsive traits across Portuguese youths.

The associations between the five factors the SUPPS-P among the two samples showed mostly moderate to large statistically significant positive associations, although the association between sensation seeking and lack of perseverance was smaller. Importantly, the associations tended to be statistically stronger among males than among females. Internal consistency values for the SUPPS-P across the male and female samples generally indicated good reliability in both groups. However, Cronbach’s alpha for the sensation seeking dimension was low in the male sample. The remaining four dimensions presented good to very good values, especially when considering the Omega coefficient results. The recommended mean inter-item correlations and corrected item-total correlations values mostly revealed that the homogeneity among the items was adequate [55]. However, the mean inter-item correlations of the lack of premeditation and positive urgency dimensions were above the recommended 0.50 cutoff for the male sample, indicating some excessive homogeneity.

The convergent validity of the SUPPS-P dimensions among the male and female samples with measures of psychopathic traits, namely dark triad traits, self-reported delinquency, aggression, and conduct disorder symptoms showed mostly positive moderate to large statistically significant correlations. These correlations were consistent with what was expected based on previous investigations associating impulsivity to these constructs, showing the partial overlap between them (for example) [10,56,57]. The discriminant validity with the BSCS mostly showed the expected negative correlations due to non-overlapping constructs. The criterion-related validity of the dimensions of the SUPPS-P with delinquency seriousness mostly revealed moderate associations, with the highest relationships being with negative and positive urgency.

The current study does have some limitations, including the reliance on a self-report methodology, which can be affected by one’s ability and willingness to respond honestly. Additionally, the samples came from a school setting and, as such, the youth at highest risk (e.g., those in forensic settings) may not be included; thus, the potential usefulness of the SUPPS-P scale in a sample of higher impulsive risk should be examined. Future research with higher impulsive risk samples should also explore the discriminant validity of the subscales of the SUPPS-P and the potential specific utility of each one of them.

In conclusion, our Portuguese translation of the SUPPS-P worked well in both male and female students. The establishment of measurement invariance across these groups allows for direct comparisons between male and female students on the SUPPS-P traits. Given the importance of adolescence as a critical period characterized by increases in impulsive behaviors, having a short, valid, reliable, and easily administered assessment of impulsive tendencies is important and clinically impactful. The SUPPS-P can be used to identify youth at risk for maladaptive risk taking and for targeted intervention or prevention strategies. For example, a recent line of research has begun to use a modified version of Dialectical Behavior Therapy [58] in school settings [59]. This treatment, named Going for Goals, is a 9-week school-based group intervention for youth who are at risk for maladaptive risk-taking [60]. Preliminary evidence with this treatment shows strong feasibility and scalability to other school settings and significant decreases in negative and positive urgency following completion of the Going for Goals program [60]. We see a prime opportunity for the SUPPS-P to be used as a way to identify youth who might benefit from skills training interventions, such as Going for Goals. In future the ability of the SUPPS-P traits to be used in schools in this manner should be examined and to document if the traits can be changed through intervention, as suggested by Zapolski and Smith [60].

## Figures and Tables

**Table 1:** Goodness of fit indexes for the different models of the SUPPS-P.

	SB*χ*^2^/*df*	IFI	CFI	RMSEA	AIC
				(90% CI)	
Total sample					
1-factor	5.44	0.95	0.95	0.10 (0.09–0.11)	585.21
5-factor	1.71	0.98	0.98	0.04 (0.03–0.05)	−46.35
5-factor 2nd order	2.67	0.92	0.92	0.06 (0.05–0.07).	110.37
Bifactor	2.2	0.95	0.95	0.05 (0.04–0.06)	30.32
Male sample					
1-factor	3.5	0.97	0.97	0.10 (0.09–0.11)	255.51
5-factor	1.39	0.97	0.97	0.04 (0.03–0.05)	−97.79
5-factor 2nd order	1.54	0.96	0.96	0.05 (0.03–0.06)	−75.86
Bifactor	1.2	0.99	0.99	0.03 (0.00–0.04)	−110.81
Female sample					
1-factor	3.7	91	0.9	0.11 (0.10–0.12)	288.34
5-factor	1.73	0.91	0.9	0.06 (0.05–0.07)	−42.95
5-factor 2nd order	1.13	0.98	0.98	0.03 (0.00–0.04)	−142.29
Bifactor	2.49	0.84	0.83	0.08 (0.07–0.10)	68.26

Note: SUPPS-P = Short UPPS-P impulsive behavior scale; SB*χ*^2^ = Satorra-Bentler Chi-square; *df* = degrees of freedom; IFI = Incremental Fit Index; CFI = Comparative Fit Index; RMSEA (90% CI) = Root Mean Square Error of Approximation (90% Confidence Interval).

**Table 2:** Loadings for the five-factor structure of the SUPPS-P.

T/M/F	Factor 1	Factor 2	Factor 3	Factor 4	Factor 5
Item 6	0.71/0.67/0.50				
Item 8	0.78/0.76/0.61				
Item 13	0.83/0.80/0.76				
Item 15	0.70/0.67/0.67				
Item1		0.69/0.52/0.58			
Item 4		0.81/0.72/0.71			
Item 7		0.81/0.76/0.67			
Item 11		0.78/0.67/0.65			
Item 2			0.80/0.71/0.66		
Item 5			0.83/0.78/0.64		
Item 12			0.83/0.82/0.65		
Item 19			0.82/0.82/0.70		
Item 9				0.91/0.89/0.79	
Item 14				0.81/0.75/0.75	
Item 16				0.46/0.29/0.49	
Item 18				0.44/0.31/0.46	
Item 3					0.80/0.77/0.61
Item 10					0.80/0.77/0.68
Item 17					0.84/0.78/0.78
Item 20					0.85/0.87/0.73

Note: SUPPS-P = Short UPPS-P impulsive behavior scale; T/M/F = Total sample/Male sample/Female sample; Factor 1 = Negative urgency; Factor 2 = Lack of perseverance; Factor 3 = Lack of premeditation; Factor 4 = Sensation seeking; Factor 5 = Positive urgency.

**Table 3:** Tests for invariance of the SUPPS-P goodness of fit statistics.

Model	SB*χ^2^*(*df*)	ΔS-B*χ^2^*(*df*)	CFI	RMSEA (90% C.I.)
1. Configural model (no constraints)	437.98 (320)	--	0.99	0.04 (0.03–0.05)
2. Metric (weak) invariance	455.80 (335)	15.49 (15)	0.99	0.04 (0.03–0.05)
3. Scalar (strong) invariance	490.13 (350)	52.95 (30) *	0.99	0.04 (0.03–0.05)

Note: SB*χ^2^*(*df*) = Satorra-Bentler Chi-square (degrees of freedom); CFI = Comparative Fit Index; RMSEA = Root Mean Square Error of Approximation; C.I. = confidence interval. * p < 0.05.

**Table 4:** Pearson correlation matrixes.

M/F	Negative Urgency	Lack of Perseverance	Lack of Premeditation	Sensation Seeking	Positive Urgency
Negative urgency	1				
Lack of perseverance	**0.52/0.36**	1			
Lack of premeditation	**0.72/0.58**	**0.65/0.60**	1		
Sensation seeking	**0.64/0.44**	**0.30/0.22**	**0.57/0.46**	1	
Positive urgency	**0.87/0.68**	**0.55/0.39**	**0.76/0.59**	**0.70/0.62**	1

Note: SUPPS-P = Short UPPS-P impulsive behavior scale. Bold values were statistically significant at 0.01. Underlined values were statistically significant (*p* < 0.05) between males and females using Fisher *z-*tests (two-tailed).

**Table 5:** Internal consistency of the SUPPS-P.

M/F	Negative Urgency	Lack of Perseverance	Lack of Premeditation	Sensation Seeking	Positive Urgency
Alpha	0.81/0.73	0.77/0.75	0.86/0.76	0.86/0.76	0.87/0.80
Omega	0.85/0.79	0.85/0.85	0.91/0.84	0.91/0.84	0.90/0.85
MIIC	0.52/0.40	0.45/0.42	0.61/0.44	0.61/0.44	0.62/0.49
CITCR	0.58–0.70/0.43–0.61	0.47–0.62/0.50–0.59	0.66–0.74/0.51–0.58	0.66–0.74/0.51–0.58	0.70–0.77/0.55–0.65

Note: SUPPS-P = Short UPPS-P impulsive behavior scale; Alpha = Cronbach’s alpha; Omega = Omega coefficient; MIIC = Mean inter-item correlation; CITCR = Corrected item-total correlation range.

**Table 6:** Convergent, discriminant and criterion validity of the SUPPS-P.

M/F	Negative Urgency	Lack of Perseverance	Lack of Premeditation	Sensation Seeking	Positive Urgency
YPI-Tri-S total	**0.77/0.57**	**0.49/0.38**	**0.71/0.58**	**0.70/0.56**	**0.85/0.63**
YPI-Tri-S Boldness	**0.68/0.50**	**0.44/0.35**	**0.66/0.52**	**0.63/0.52**	**0.76/0.57**
YPI-Tri-S Disinhibition	**0.74/0.48**	**0.46/0.34**	**0.65/0.49**	**0.66/0.49**	**0.80/0.55**
YPI-Tri-S Meanness	**0.76/0.54**	**0.48/0.34**	**0.71/0.57**	**0.69/0.50**	**0.84/0.60**
DD total	**0.75/0.51**	**0.43/0.29**	**0.67/0.44**	**0.67/0.51**	**0.83/0.58**
DD Machiavellianism	**0.70/0.48**	**0.44/0.30**	**0.62/0.43**	**0.58/0.49**	**0.74/0.53**
DD Psychopathy	**0.76/0.57**	**0.52/0.38**	**0.73/0.54**	**0.66/0.50**	**0.85/0.64**
DD Narcissism	**0.50/0.25**	0.16/0.08	**0.38** /0.16	**0.51/0.29**	**0.57/0.29**
AHSRD total	**0.73/0.51**	**0.43/0.27**	**0.66/0.51**	**0.57/0.45**	**0.77/0.54**
AHSRD Non-violent	**0.75/0.52**	**0.45/0.28**	**0.67/0.52**	**0.60/0.45**	**0.79/0.54**
AHSRD Violent	**0.60/0.22**	**0.32/** 0.07	**0.55/0.28**	**0.45/0.23**	**0.65/0.29**
PCS-20 total	**0.69/0.42**	**0.43/0.21**	**0.66/0.40**	**0.53/0.31**	**0.70/0.37**
PCS-20 Reactive-Relational	**0.40/0.33**	**0.25/0.23**	**0.42/0.27**	**0.30/0.24**	**0.40/0.31**
PCS-20 Proactive-Relational	**0.58/0.28**	**0.34** /0.13	**0.56/0.29**	**0.42/0.19**	**0.56/0.23**
PCS-20 Reactive-Overt	**0.68/0.38**	**0.43** /0.13	**0.64/0.36**	**0.55/0.31**	**0.70/0.32**
PCS-20 Proactive-Overt	**0.71/0.43**	**0.46/0.24**	**0.68/0.42**	**0.56/0.29**	**0.73/0.41**
CDS total	**0.75/0.46**	**0.45/0.33**	**0.66/0.47**	**0.59/0.41**	**0.78/0.47**
BSCS total	**−0.79/−0.61**	**−0.61/−0.56**	**−0.82/−0.71**	**−0.63/−0.51**	**−0.82/−0.67**
GDSC	**0.71/0.46**	**0.42/0.25**	**0.64/0.46**	**0.57/0.40**	**0.74/0.45**

Note: SUPPS-P = Short UPPS-P impulsive behavior scale; YPI-Tri-S = Youth Psychopathic Traits Inventory Triarchic-Short; DD = Dirty Dozen; AHSRD = Add Health Self-Report Delinquency scale; PCS-20 = Brief Peer Conflict Scale; CDS = Conduct Disorder Screener; BSCS = Brief Self-Control Scale; GDSC = General Delinquency Seriousness Classification. Bold values were statistically significant at 0.01. Underlined values were statistically significant (*p* < 0.05) between males and females using Fisher *z*-tests (2-tailed).

## Data Availability

Data can be available to consultation when request to the corresponding author and with permission of the participants of the study.
